# Utilizing Artificial Intelligence to create narrative literature reviews

**DOI:** 10.31744/einstein_journal/2026RW1165

**Published:** 2026-03-13

**Authors:** Auro del Giglio, Mateus Uérlei Pereira da Costa

**Affiliations:** 1 Centro Universitário FMABC Santo André SP Brazil Centro Universitário FMABC, Santo André, SP, Brazil.; 2 Sociedade Brasileira de Oncologia Clínica São Paulo SP Brazil Sociedade Brasileira de Oncologia Clínica, São Paulo, SP, Brazil.

**Keywords:** Medical writing, Artificial Intelligence, Review literature as topic, Research design

## Abstract

This study explores the potential impact of Artificial Intelligence on narrative literature reviews in academic research. The literature review process involves finding, analyzing, and synthesizing relevant literature and is crucial for situating new research within existing knowledge. The integration of Artificial Intelligence tools, specifically Large Language Models such as the Generative Pre-Trained Transformer series, can significantly improve the efficiency and effectiveness of this process. This paper outlines the steps involved in conducting a literature review and examines how Artificial Intelligence tools can aid in identifying research gaps, organizing and analyzing retrieved articles, and writing the review. In the literature review process, we provide examples of both free and commercially available Artificial Intelligence software to demonstrate their potential applications.

## INTRODUCTION

A literature review is a scholarly process that involves identifying, analyzing, summarizing, and organizing relevant literature to provide a coherent overview of what is known, unknown, and contested in a particular field.^(1,2)^ In academic research, a comprehensive literature review is essential for gaining insight into the latest advancements in treatments and procedures.^(1,2)^ It is the cornerstone for preparing scientific articles, grant proposals, and doctoral theses. This endeavor, which is crucial for situating new research within the context of existing knowledge, can be primarily categorized into systematic and narrative (or traditional) reviews.^(1,2)^

Systematic reviews are characterized by their methodical and structured approach, focusing on answering specific research questions through comprehensive search strategies, explicit inclusion and exclusion criteria, and systematic presentation of study findings.^(3)^ In contrast, narrative reviews adopt a more flexible and interpretative approach while also aiming to summarize and synthesize the existing literature, allowing for a broader exploration of the topic without the stringent methodological constraints of systematic reviews.^(1,2,4)^ Given their expansive nature, narrative reviews are particularly adept at providing comprehensive overviews of the field, identifying theoretical gaps, and highlighting areas for future research.

The advent of Artificial Intelligence (AI) tools in recent years, powered by Large Language Models (LLMs) such as the Generative Pre-trained Transformer (GPT) series from OpenAI, is a groundbreaking advancement that can potentially enhance the literature review process.^(5,6)^ Large Language Models, which are sophisticated AI technologies developed through training on vast amounts of textual data, can execute various linguistic functions, including writing, translation, and summarization. These models have shown exceptional skills in understanding and generating natural language, demonstrating how LLMs can transform the craft of scientific writing, particularly in the context of narrative literature reviews.^(5–8)^

Given the critical role of literature reviews in the meticulous planning and reporting of research, the use of AI tools to accelerate associated tasks holds promise for significantly reducing the time researchers spend on these activities. This study explores how AI-powered tools can support each step of the narrative literature review process, potentially revolutionizing the efficiency and effectiveness of scientific literature. We also provide examples and elaborate on the use of freely available AI software (Tables 1 and 2).

**Table 1:** Selected applications, respective weblinks, and a brief description of their features

Software name	Link	Description of features applicable to scientific writing*
ChatGPT	OpenAI	Generates research gaps, creates outlines, drafts paper sections (*e.g*., introduction, discussion), suggests language tone adjustments, and enhances readability. Can also critique papers similar to a peer review
Research Rabbit	Research Rabbit	Identifies related papers, authors, and topics via citation and co-authorship analysis. Facilitates literature exploration through a visual interface and integrates with Zotero for easy citation management
Semantic Scholar	Semantic Scholar	Uses natural language processing and machine learning to search for papers, understand content within field context, and rank papers by relevance
Elicit	Elicit	Uses advanced search strategies to find and tabulate relevant papers. Provides a unique interface for efficiently sorting literature findings
Zotero	Zotero	Free Reference Management System (RMS) for collecting, organizing, citing, and sharing research sources. Features include browser integration, PDF annotation, tagging, citation, bibliography management, and collaboration support
Mendeley	Mendeley	Web-based RMS similar to Zotero, with integration into online Microsoft Word for cloud-based citation and bibliography management
TinyWow	TinyWow	Provides PDF summarization tools for quick screening of articles to assess relevance
Scribbr	Scribbr	Offers PDF summarization to help manage large volumes of literature by providing concise article summaries
Quillbot	Quillbot	Summarizes scientific articles for initial screenings to determine relevance and importance for narrative reviews
Grammarly	Grammarly	AI-powered writing assistant for spelling, grammar, and style to ensure clarity and correctness. Integrates with word processors for real-time assistance
Jenni.ai	Jenni.ai	Combines spelling/grammar correction, text improvement, and bibliographic citation assistance. Enhances writing by suggesting sentence development and integrating writing aids
Yomu	Yomu.ai	Integrates writing aids like text improvement and bibliographic reference help. Supports the writing process through features similar to Jenni.ai
Claude	Claude	Advanced conversational AI for writing assistance, summarization, and brainstorming in scientific writing. Known for safety alignment and long-context handling
Perplexity	Perplexity	AI-powered search engine delivering concise answers and sources, helpful for retrieving up-to-date references during literature reviews
DeepSeek Chat	DeepSeek	Large Language Model designed for deep reasoning, code-based tasks, scientific Q&A, problem-solving, and drafting technical content
Llama/Mistral via DuckDuckGo/Groq	Llama	DuckDuckGo AI Chat platform offering access to Llama and Mistral via Groq, useful for quick scientific queries and summarizations with fast response times

* Several of these are powered by Artificial Intelligence.

**Table 2:** Steps required to conduct a narrative review

Step	Description	Software used
Research Gap Identification	Identifying areas where knowledge is lacking within a specific field. This is the foundation for any narrative review	ChatGPT
Creating an Outline	Developing a structured framework for the review, outlining major themes, findings, and the logical flow of the review	ChatGPT
Literature Search	Searching for relevant articles, books, and other sources to include in the review. This involves using specific keywords and search criteria to find pertinent literature	ChatGPT, Research Rabbit, Semantic Scholar, Elicit
Data Organization	Organizing the retrieved literature and data in a systematic way for easy access and analysis	Zotero, Mendeley
Studying and Annotating Important References	In-depth reading and annotation of key literature to understand the field, identify controversies, and gather insights for future research	Zotero, Mendeley
Review Drafting	Writing the review based on the organized and annotated literature, synthesizing findings, and discussing their implications	ChatGPT, Grammarly, Jenni.ai, Google Docs (word processor)
Citation Management	Properly citing all references used in the review to acknowledge sources and minimize plagiarism	Zotero, Mendeley

## METHODS

This narrative review examined the use of AI-powered tools in the composition of narrative reviews. We explored the literature within this domain through searches conducted in PubMed and Google Scholar databases. The search strategy involved combining the terms "narrative review" and "medicine" with variations of "AI tools," "artificial intelligence," or "machine learning," along with "writing." In PubMed, Medical Subject Heading (MeSH) terms are used for greater precision. There were no date limits in the search. A total of 40 articles were retrieved from PubMed, whereas the first two hundred references in Google Scholar were retrieved.

The references most related to our review were then uploaded to Research Rabbit software and later elicited to identify other articles through a semantic strategy.

We also reviewed the lists of selected articles and web pages of the software selected for inclusion in the review.

We excluded articles dealing with open-source software that required prior training and articles dealing with software to write systematic reviews and meta-analyses. Finally, we selected the articles that were most relevant to our objectives.

Because this review was non-systematic, the articles selected for inclusion and citation were those deemed the most relevant by the authors. Therefore, the search strategy and reference list described herein may not be reproducible. In Table 1S, Supplementary Material, we present all the saved entries in the Zotero database used to draft this article.

## RESULTS AND DISCUSSION

The results and discussion of this study are divided into seven sections based on the steps involved in the literature review supported by AI. Additionally, we briefly discuss the limitations of using AI in scientific writing.

### Steps required to conduct a narrative review

Writing narrative medical reviews involves a structured approach. We started by searching for emerging trends in the medical field in order to identify one or more research gaps suitable for development in a review. Next, we provided an outline of this review.^(1,2,4)^ This outline serves as the basis for a literature search using strategic keywords to find relevant articles for each part of the article.

The next steps included developing inclusion and exclusion criteria to screen and select pertinent studies, extracting and organizing data from these studies for analysis, and synthesizing the information to identify common findings and gaps. Writing the review entails drafting key sections such as the introduction, which highlights the importance of the review; the methodology, which describes the approach for selecting and analyzing studies; and the discussion, which critically examines the findings. The last step involves managing references and citations, and ensuring adherence to specific citation styles, such as the Vancouver style (Table 2).

### Identifying research gaps using AI

A research gap pertains to the inadequacy, insufficiency, incompleteness, contradictions, or obsolescence of the existing literature, theories, or empirical evidence within a specific field of study. This signifies a void in current knowledge or comprehension, indicating the need for further investigation.^(9,10)^ Research gaps can arise because of several factors such as constraints in past research methodologies, phenomena yet to be explored, unanswered queries from preceding studies, and changes in societal or technological contexts that warrant reassessment of prior findings.

While it may not be imperative to undertake a comprehensive search for a research gap when conducting a narrative review, if the identification of such a gap has already been suggested by a mentor or arisen from unexpected research outcomes, for those seeking to create a narrative review who have not yet identified a research gap, there are several ways through which one may use AI to aid in the identification of a viable research gap.

One viable approach is to identify recent publications in the field of interest within the last six to twelve months in reputable peer-reviewed journals. A thorough examination of the discussion sections of these articles may reveal specific areas requiring further investigation, thereby identifying one or more research gaps. As elucidated in subsequent sections of this literature review, AI tools can facilitate the identification of relevant literature.

**Figure 1:**
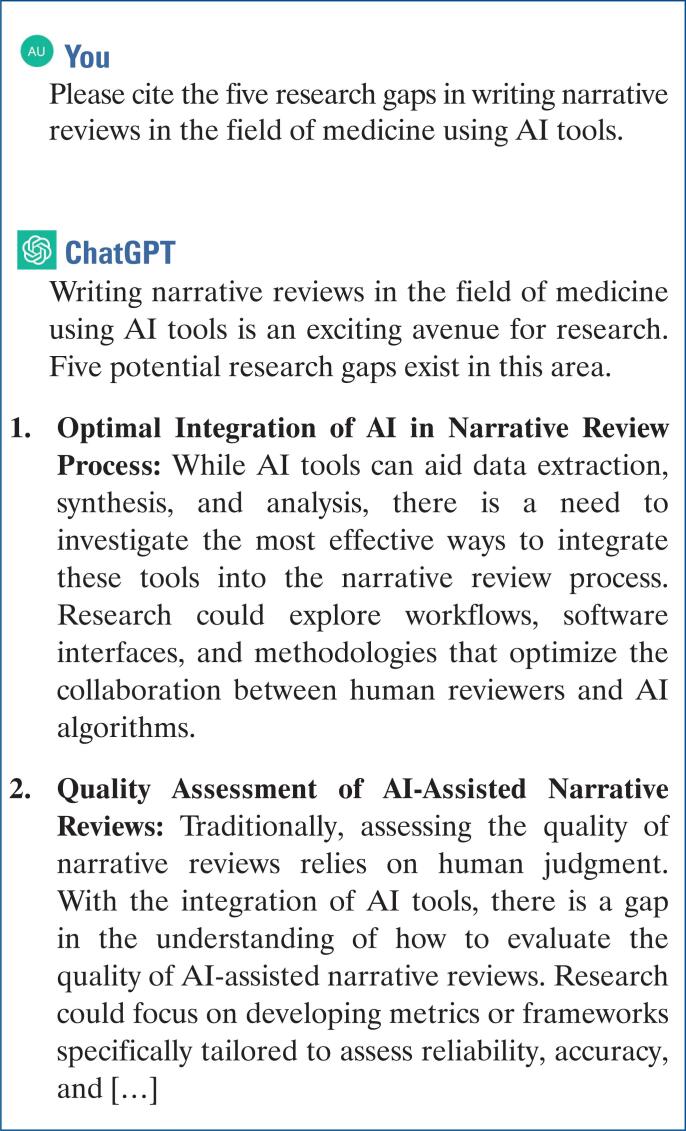
ChatGPT can be used to help find a research gap

An alternative approach involves engaging with LLM ChatGPT to identify potential research gaps within a specific area of study (Figure 1). Once a research gap has been identified as the subject of the narrative review, the subsequent step involves conducting a thorough literature search and evaluating the retrieved papers as potential sources of material for review.

### Creating outlines for reviews using AI tools

**Figure 2:**
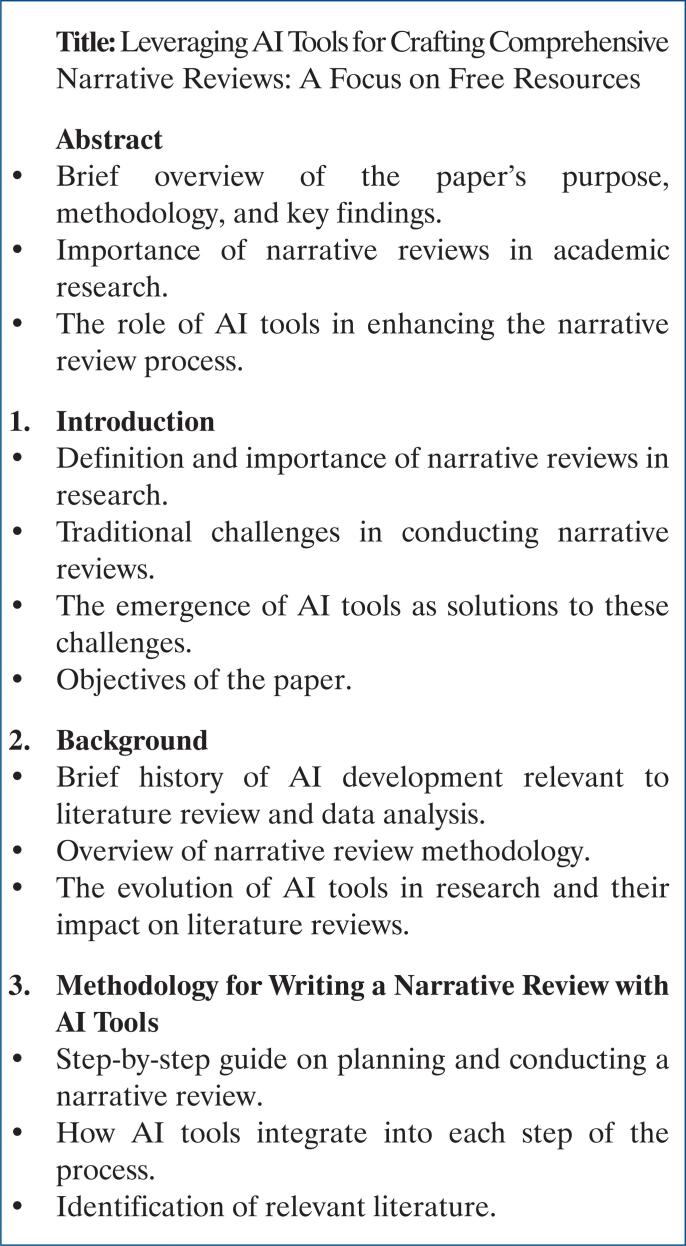
Schematic representation of the review outline generated by ChatGPT-3.5

To optimize the writing of a narrative review, one must follow an outline containing all parts of the review to be written in logical order (Figure 2). A direct way of creating an outline for a narrative review is to use ChatGPT. Another method is to organize all the references retrieved thus far into different subjects through tags in the Reference Management System (RMS) (see below), which will be organized later in logical order and used to generate an outline. Generally, both strategies can be integrated to derive a definitive outline of a review.

### Identifying pertinent papers in the literature using AI tools

**Figure 3:**
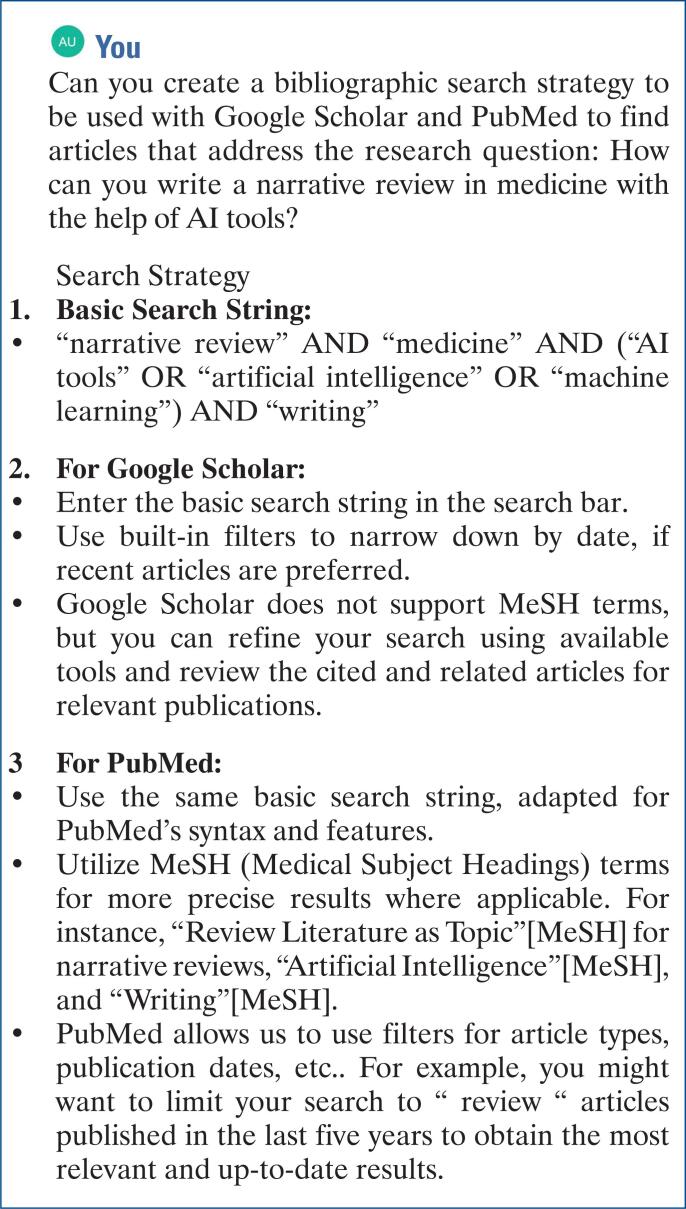
Optimizing search strategies for PubMed and Google Scholar Databases with assistance from ChatGPT-3.5

A classic approach to finding relevant papers is to search specific databases, such as PubMed and Google Scholar. ChatGPT can help create a specific search strategy for these databases (Figure 3).

**Figure 4:**
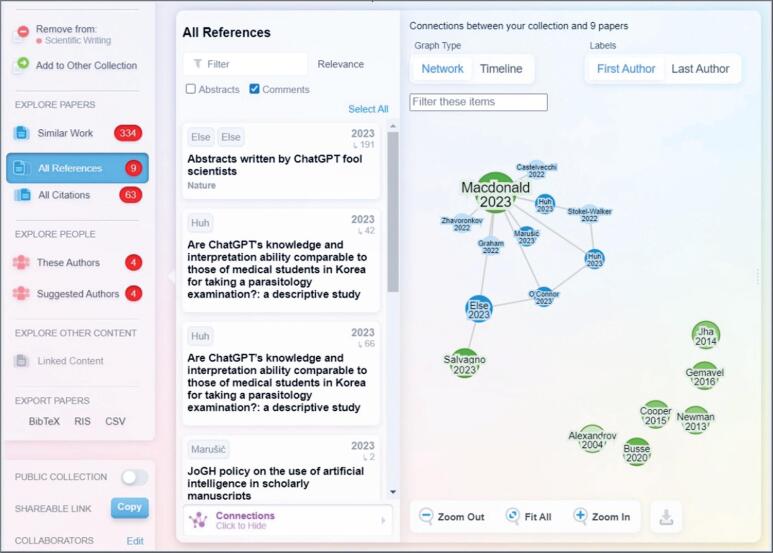
Research Rabbit visualization of article relationships: a graphical representation revealing interconnections through keywords, citations, and co-authorship

Another method is to use AI tools, such as Research Rabbit, which can directly scan the literature, starting with a paper or topic initially selected by the researcher. Research Rabbit identifies related papers, relevant authors who have published in the area, and other relevant topics. Instead of using keywords, the system examines citations and co-authorships to identify the connections between different works. Furthermore, users can explore a graph of connected papers (Figure 4), diving into related topics, or following chains of citations to uncover the literature most relevant to their research interests. The visual interface of the platform enhances interactive exploration, making it easy to observe how different papers are connected. An additional advantage of Research Rabbit is its integration with Zotero, a powerful and free RMS.^(11)^ The papers retrieved by Research Rabbit are directly exportable to Zotero for storage and are later cited in the narrative review.

Other AI tools, such as Semantic Scholar, can search for papers using natural language processing. These semantic strategies enable the system to understand the content of papers in the context of their field of study and use machine learning to rank papers according to their relevance. Elicit uses search strategies such as Semantic Scholar to find articles and tabulates the results of the most relevant papers. There are other online AI-powered resources for literature reviews, such as Perplexity (https://www.perplexity.ai/) and Claude (www.claude.ai), which can complement searches using the tools described above.

The literature research step may need to be repeated several times during the drafting of the manuscript, as parts of the outline may not have sufficient references already selected and stored in the RMS. Therefore, one may need to search again, targeting a specific subsection of the review for which more content is lacking. Furthermore, searching for bibliographies of the selected papers and browsing through important recent meeting abstracts may unravel additional references for inclusion in the review (Tables 1 and 2).

### Organizing and studying the articles retrieved using AI tools

Zotero is a free RMS^(11)^ that uses a plugin for web browsers such as Google Chrome, Microsoft Edge, or Firefox. It allows downloading of scientific articles and their respective PDF files and metadata from the Internet, such as authors, publication dates, journals, and abstracts. The retrieved papers are stored in specific libraries created by the user. The authors can then highlight the essential parts of the text in the PDF files to tag and store notes in Zotero's PDF editor. Comments can also be added to notes. The text in these notes can be searched and exported to Microsoft Word and Google Docs. Tagging these notes facilitates the search while writing the review text. For instance, if one tags a note as "Introduction" or "Discussion," this may allow one to easily retrieve all the notes whose content could help write each one of these sections. Notably, annotating and highlighting selected parts of a text is a valuable way to study and summarize scientific articles, which are critical steps in the literature review process. Exportable notes can help draft parts of a review text and always acknowledge their sources to avoid plagiarism.

Zotero also allows citation references in the text of desktop versions of Microsoft Word and Google Docs using a plugin downloadable from the Zotero website. This plugin is compatible with Microsoft Word versions of Windows and MAC operating systems. For Google Docs, the plugin operates on the cloud. The system also allows the bibliography of the narrative review to be created automatically based on citations inserted into the text in distinctive styles (e.g. Vancouver). Furthermore, the bibliography can be updated automatically as new citations are added or removed from a paper. In addition, researchers can collaboratively use Zotero.

Elsevier's Mendeley is a web-based free RMS with functionalities similar to Zotero. Unlike Zotero, Mendeley can be used with an online version of Microsoft Word.

Another valuable resource is the availability of free PDF file summarizers such as TinyWell and Scribbr, as well as other commercial solutions such as Quillbot. These tools can summarize scientific articles in PDF files, and their outputs can be stored in Zotero or Mendeley notes (Table 1). However, the quality of these summaries may not be as good as that of their respective author abstracts. Furthermore, the automatic process of summarizing an article using an AI tool does not substitute for the learning process involved in reading the original paper, manually selecting critical parts of the text, and creating commentaries. Therefore, PDF summarizers may help review articles that have not yet been read to determine if they should be read in full.

### Writing reviews using AI

This part of the process begins with the outline. Each part of the outline is written based on notes taken within the RMS system while retrieving and organizing bibliographic references. One can often import text from these notes to a word processor and, by rephrasing the imported texts and citations of their sources, build each part of the review required to complete the outline.

Several AI-powered tools, including spelling and grammar correctors, can assist authors during the writing phase. Examples include the editor of Microsoft Word, spelling and grammar checks of Google Docs, and software such as Grammarly, which can be integrated into Word processors. In addition, ChatGPT can create drafts for all parts of a paper, including the Title, Abstract, Introduction, and Discussion. ChatGPT can modify the text to adjust the tone, making it more academic, or suggesting changes to enhance the flow and readability of the text. ChatGPT can also criticize the paper or any of its sections, making valuable suggestions as a reviewer would upon evaluating the article before publication.^(5,6)^

Commercial software such as Jenni.ai and Yomu offer spelling and grammar corrections, text improvement suggestions, and help with bibliographic citations. In addition, these platforms can create parts of the text while writing a phrase by suggesting diverse ways to develop a sentence. These platforms can be costly and, in our view, improve the writing process compared with the use of a Word Processor, an RMS, and ChatGPT separately. However, integrating all these features into a single platform may accelerate the writing process more effectively than using different software programs separately (Tables 1 and 2).

### Limitations of AI for scientific writing

Although AI-powered tools may be several times more efficient than humans for various tasks, such as researching literature, summarizing articles, and creating bibliographies, they lack creativity, which is an exclusively human characteristic.^(12)^ Regarding the writing of narrative reviews, author insights based on the reviewed literature are of paramount importance if the text is to be useful to other researchers in the field. A narrative review that only cites and briefly summarizes other articles without creating new and original ways of understanding this large amount of information is not useful. Therefore, in our view, authors should not depend solely on AI tools to summarize important articles, but instead read them thoroughly and in depth to generate newer insights regarding controversies, research gaps, and future directions of development in the field. Although one can use ChatGPT to identify some of the gaps, future directions, or research in a particular field, because this LLM is built and trained on existing information, the novelty of these outputs is questionable and potentially biased.^(12)^ It is also important to emphasize the importance of prompt engineering to obtain better and more precise outputs from LLMs. Prompt engineering is the practice of strategically designing text inputs (prompts) for an AI model to elicit desired responses, control tone or style, improve factual accuracy, or align outputs with a specific task or goal. The more specific and rich the context information of a prompt, the better the output that the LLM will generate to answer a specific request. Furthermore, prompt phrasing or temperature settings can influence LLM outputs; therefore, these parameters should be considered when designing prompts.

It is important to note that the rapid evolution of LLMs may provide authors with more powerful resources that may impact the quality of the AI output for the same input. For example, GPT-4 performed significantly better than GPT-3.5 in several ways.

Another concern is that AI-generated reviews may add little value to their respective fields, serving only to satisfy the "publish or perish" requirements of academic life. Scientific writing should aim to contribute novel information or conceptual innovations to a particular field of knowledge. Therefore, using AI as a tool rather than as a brain is essential for improving writing efficiency while maintaining its usefulness in advancing scientific knowledge.^(13)^ An additional limitation of AI is the occurrence of hallucinations that can produce inaccurate results. Therefore, the authors should screen all AI-generated outputs for potential errors.^(5)^ Bibliographic references need to be individually checked for accuracy, as well as for the content generated by LLMs. Inaccuracies that require rewriting or complete reformulation may occur.

Another limitation that may arise from incorrect information obtained through LLMs is the clinical risk of using inaccurate AI answers, particularly in specialties such as cardiology, where decision making is high.

Despite these limitations, the use of AI, whenever employed, needs to be transparently acknowledged in writing, specifying each task for which it was utilized and the parts of the paper in which it was employed. This way, AI can be ethically incorporated into scientific writing.^(12)^

## CONCLUSION

The integration of Artificial Intelligence into narrative literature reviews has immense potential for revolutionizing the efficiency and effectiveness of this critical research endeavor. With the aid of Artificial Intelligence-powered tools, researchers can quickly identify relevant research gaps, generate outlines, conduct literature searches, and organize retrieved articles. Additionally, Artificial Intelligence can aid in the writing process. Artificial Intelligence not only saves time but also enhances the quality of the review by suggesting appropriate corrections to the text.

However, it is essential to note that Artificial Intelligence tools should not replace the vital process of critically reading and analyzing scientific literature. Researchers should not expect original insights, advanced understanding of research gaps, or the generation of feasible and appropriate solutions from Artificial Intelligence trained on previously available information. Instead, they should be used as complementary aids to improve the overall writing of literature reviews. As Artificial Intelligence technology advances, researchers can look forward to streamlining their work and producing high-quality narrative literature reviews more efficiently.

## Data Availability

Data are available to reviewers upon request.
