# Supplementary material for: Utilizing Artificial Intelligence to create narrative literature reviews
Source: Einstein (Sao Paulo). 2026 Mar 13;24:eRW1165. doi: 10.31744/einstein_journal/2026RW1165 (PMC13128250; doi:10.31744/einstein_journal/2026RW1165)
Supplement: Supplementary Material [file 2317-6385-eins-24-eRW1165-suppl01.pdf]

SUPPLEMENTARY MATERIAL

Utilizing Artificial Intelligence to create narrative literature reviews

Auro del Giglio, Mateus Uérlei Pereira da Costa

DOI: 10.31744/einstein_journal/2026RW1165

Table 1S. Entries in the Zotero database

Zotero Library employed
"8 Best AI Tools for Research in 2023". Acesso em 17 de março de 2024. https://wordvice.ai/blog/8-best-ai-tools-for-researchers.
"(86) Top (FREE) Ai Tools for Research and Academic Writing 2023 | LinkedIn". Acesso em 17 de março de 2024. https://www.linkedin.com/pulse/top-free-ai-tools-research-academic-writing-2023-anik-barua/.
Ahaley, Shital Sarah, Ankita Pandey, Simran Kaur Juneja, Tanvi Suhane Gupta, e Sujatha Vijayakumar. "ChatGPT in Medical Writing: A Game-Changer or a Gimmick?" Perspectives in Clinical Research, 15 de novembro de 2023. https://doi.org/10.4103/picr.picr_167_23.
Altmäe, Signe, Alberto Sola-Leyva, e Andres Salumets. "Artificial Intelligence in Scientific Writing: A Friend or a Foe?" Reproductive BioMedicine Online 47, no 1 (julho de 2023): 3–9. https://doi.org/10.1016/j.rbmo.2023.04.009.
AskYourPDF. "7 Best AI for Research Paper Writing", 21 de dezembro de 2023. https://askyourpdf.com/blog/best-ai-for-research-paper-writing.
BMnJL2UTndtU. "AI Tools for Science Writing: Why, How, When, and When Not To". SciTechEdit International (blog), 6 de novembro de 2023. https://scitechedit.com/using-ai-tools-for-science-writing/.
Buchkremer, Rüdiger, Alexander Demund, Stefan Ebener, Fabian Gampfer, David Jagering, Andreas Jürgens, Sebastian Klenke, et al. "The Application of Artificial Intelligence Technologies as a Substitute for Reading and to Support and Enhance the Authoring of Scientific Review Articles". IEEE Access, 2019. https://doi.org/10.1109/access.2019.2917719.
Buriak, Jillian M., Deji Akinwande, Natalie Artzi, C. Jeffrey Brinker, Cynthia Burrows, Warren C. W. Chan, Chunying Chen, et al. "Best Practices for Using AI When Writing Scientific Manuscripts: Caution, Care, and Consideration: Creative Science Depends on It". ACS Nano 17, no 5 (14 de março de 2023): 4091–93. https://doi.org/10.1021/acsnano.3c01544.
Chen, Tzeng-Ji. "ChatGPT and Other Artificial Intelligence Applications Speed up Scientific Writing". Journal of the Chinese Medical Association 86, no 4 (abril de 2023): 351–53. https://doi.org/10.1097/JCMA.0000000000000900.
Cronin, Patricia, Frances Ryan, e Michael Coughlan. "Undertaking a Literature Review: A Step-by-Step Approach". British Journal of Nursing 17, no 1 (1o de janeiro de 2008): 38–43. https://doi.org/10.12968/bjon.2008.17.1.28059.
EdrawMind. "The Leading AI Research Paper Writers You Can Rely On". Acesso em 5 de março de 2024. https://edrawmind.wondershare.com/ai-features/research-paper-ai-writer.html.
euronews. "These AI Tools Could Help Boost Your Academic Research", 20 de janeiro de 2024. https://www.euronews.com/next/2024/01/20/best-ai-tools-academic-research-chatgpt-consensus-chatpdf-elicit-research-rabbit-scite.
Gao, Catherine A., Frederick M. Howard, Nikolay S. Markov, Emma C. Dyer, Siddhi Ramesh, Yuan Luo, e Alexander T. Pearson. "Comparing Scientific Abstracts Generated by ChatGPT to Original Abstracts Using an Artificial Intelligence Output Detector, Plagiarism Detector, and Blinded Human Reviewers". Preprint. Scientific Communication and Education, 27 de dezembro de 2022. https://doi.org/10.1101/2022.12.23.521610.
Giglio, Auro del, e Mateus Uerlei Pereira da Costa. "The use of artificial intelligence to improve the scientific writing of non-native english speakers". null, 2023. https://doi.org/10.1590/1806-9282.20230560.
Gilat, Ron, e Brian J. Cole. "How Will Artificial Intelligence Affect Scientific Writing, Reviewing and Editing? The Future is Here …". Arthroscopy: The Journal of Arthroscopy And Related, 2023. https://doi.org/10.1016/j.arthro.2023.01.014.
Golan, Roei, Rohit Reddy, Akhil Muthigi, e Ranjith Ramasamy. "Artificial intelligence in academic writing: a paradigm-shifting technological advance". Nature reviews. Urology, 2023. https://doi.org/10.1038/s41585-023-00746-x.
Green, Bart N., Claire D. Johnson, e Alan Adams. "Writing narrative literature reviews for peer-reviewed journals: secrets of the trade". Journal of Chiropractic Medicine 5, no 3 (2006): 101–17. https://doi.org/10.1016/S0899-3467(07)60142-6.
"Here's What Happens When ChatGPT Writes a Scientific Article | TIME". Acesso em 5 de março de 2024. https://time.com/6695917/chatgpt-ai-scientific-study/.
Huespe, Ivan A., Jorge Echeverri, Aisha Khalid, Indalecio Carboni Bisso, Carlos G. Musso, Salim Surani, Vikas Bansal, e Rahul Kashyap. "Clinical Research With Large Language Models Generated Writing—Clinical Research with AI-Assisted Writing (CRAW) Study". Critical Care Explorations 5, no 10 (2 de outubro de 2023): e0975. https://doi.org/10.1097/CCE.0000000000000975.
Kacena, Melissa A., Lilian I. Plotkin, e Jill C. Fehrenbacher. "The Use of Artificial Intelligence in Writing Scientific Review Articles". Current Osteoporosis Reports 22, no 1 (fevereiro de 2024): 115–21. https://doi.org/10.1007/s11914-023-00852-0.
Kammer, Michael N. "The Role of Artificial Intelligence in Scientific Writing". Journal of Clinical Case Reports Medical Images and Health Sciences 3, no 3 (17 de fevereiro de 2023). https://doi.org/10.55920/JCRMHS.2023.03.001116.
King, Michael R. "The Future of AI in Medicine: A Perspective from a Chatbot". Annals of Biomedical Engineering, 2023. https://doi.org/10.1007/s10439-022-03121-w.

continue...

...Continuation

Lechien, Jérôme R., Amy Gorton, Jean Robertson, e Luigi Angelo Vaira. "Is ChatGPT-4 Accurate in Proofread a Manuscript in Otolaryngology–Head and Neck Surgery?" null, 2023. https://doi.org/10.1002/ohn.526.

Li, Pengcheng. "An Artificial Intelligence Conversational Chatbot Developed for Non-Native English Speakers". Highlights in Science, Engineering and Technology 1 (14 de junho de 2022): 97–100. https://doi.org/10.54097/hset.v1i.433.

Marina, Valerija, e Genovaite Snuviškiene. "Error Analysis of Scientific Papers Written by Non-Native Speakers of English". Transport, 2005. https://doi.org/10.3846/16484142.2005.9638031.

Meade, Matthew, William DiCiurcio, Tyler Radack, Mark Michael, e Barrett Woods. "Reference Managers". Clinical Spine Surgery 37, no 2 (1o de março de 2024): 77–78. https://doi.org/10.1097/BSD.0000000000001532.

Milian, Ricardo Diaz, Pablo Moreno Franco, William D. Freeman, e John Halamka. "Revolution or Peril? The Controversial Role of Large Language Models in Medical Manuscript Writing". null, 2023. https://doi.org/10.1016/j.mayocp.2023.07.009.

Mohanan, Saritha, e Narayanan Parameswaran. "FINER Criteria – What Does It Mean?" Cosmoderma 2 (19 de novembro de 2022): 115. https://doi.org/10.25259/CSDM_123_2022.

Parisis, Nikolaos. "Medical Writing in the Era of Artificial Intelligence", [s.d.].

Park, Michael, Erin Leahey, e Russell J. Funk. "Papers and patents are becoming less disruptive over time". Nature, 2023. https://doi.org/10.1038/s41586-022-05543-x.

Pautasso, Marco. "Ten Simple Rules for Writing a Literature Review". PLOS Computational Biology 9, no 7 (18 de julho de 2013): e1003149. https://doi.org/10.1371/journal.pcbi.1003149.

Pearce, Joshua M. "How to Perform a Literature Review with Free and Open Source Software". Acesso em 18 de março de 2024. https://doi.org/10.7275/JJHZ-SZ75.

Pegoraro, Alessandro, Kavita Kumari, Hossein Fereidooni, e Ahmad-Reza Sadeghi. "To ChatGPT, or Not to ChatGPT: That Is the Question!" arXiv, 5 de abril de 2023. http://arxiv.org/abs/2304.01487.

Perkins, Mike, e Jasper Roe. "Academic Publisher Guidelines on AI Usage: A ChatGPT Supported Thematic Analysis". F1000Research, 23 de outubro de 2023. https://doi.org/10.12688/f1000research.142411.1.

"Academic Publisher Guidelines on AI Usage: A ChatGPT Supported Thematic Analysis". F1000Research 12 (23 de outubro de 2023): 1398. https://doi.org/10.12688/f1000research.142411.1.

R, Dr Somasundaram. "Top 7 Artificial Intelligence (AI) Tools in Scientific Research". iLovePhD, 13 de março de 2024. https://www.ilovephd.com/top-7-artificial-intelligence-ai-tools-in-scientific-research/.

Salvagno, Michele, Fabio Silvio Taccone, e Alberto Giovanni Gerli. "Can Artificial Intelligence Help for Scientific Writing?" Critical Care 27, no 1 (25 de fevereiro de 2023): 75. https://doi.org/10.1186/s13054-023-04380-2.

Temsah, Omar, Samina A Khan, Yazan Chaiah, Abdulrahman Senjab, Khalid Alhasan, Amr Jamal, Fadi Aljamaan, et al. "Overview of Early ChatGPT's Presence in Medical Literature: Insights From a Hybrid Literature Review by ChatGPT and Human Experts". Cureus, 8 de abril de 2023. https://doi.org/10.7759/cureus.37281.

"The 5 Best AI Tools for Postgraduate Research", 8 de agosto de 2022. https://www.scholarcy.com/the-5-best-ai-tools-for-postgraduate-research.

"TopFREEAiToolsforResearchandAcademicWriting2023.pdf(Review) - Adobe cloud storage". Acesso em 17 de março de 2024. https://acrobat.adobe.com/id/urn:aaid:sc:VA6C2:9686b934-c074-4c22-84ce-26fe4f21fcc4.

"Wisio | Write Science Powered by AI - AI for Scientific Writing". Acesso em 5 de março de 2024. https://wisio.app/.
